# Supplementary figures and images for: Regional response of grassland productivity to changing environment conditions influenced by limiting factors
Source: PLoS One. 2020 Oct 16;15(10):e0240238. doi: 10.1371/journal.pone.0240238 (PMC7567387; doi:10.1371/journal.pone.0240238)

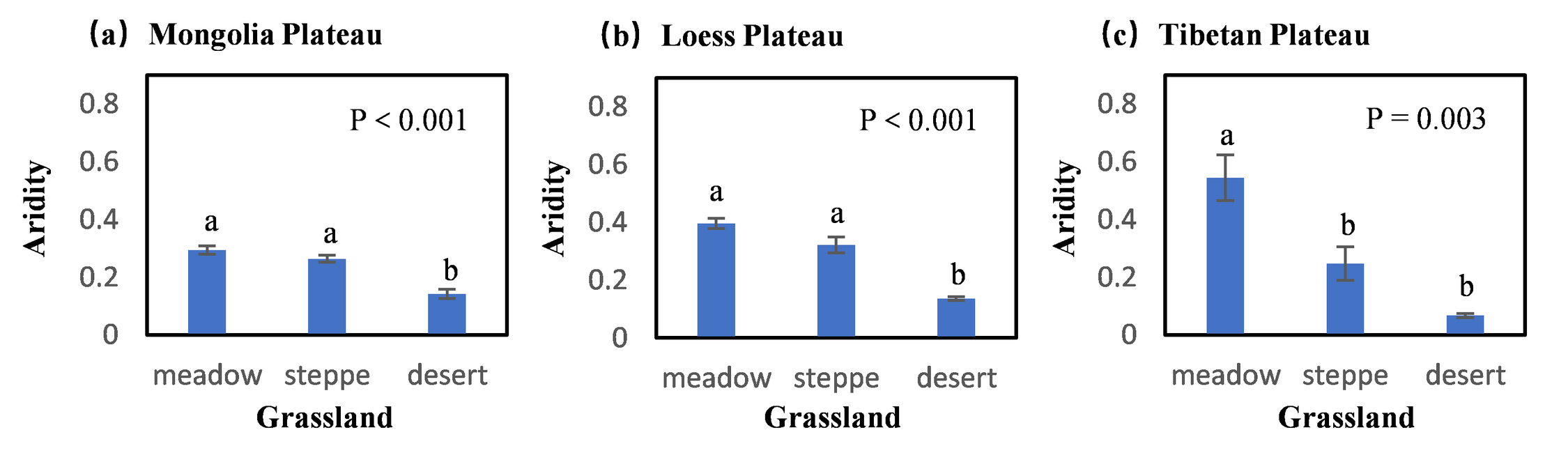

Supplement: S1 Fig — Error line represents 1 * standard error; different letters (a, b) indicate significant difference (P < 0.05). (TIF) [file pone.0240238.s001.tif]
