# Supplementary material for: Regional response of grassland productivity to changing environment conditions influenced by limiting factors
Source: PLoS One. 2020 Oct 16;15(10):e0240238. doi: 10.1371/journal.pone.0240238 (PMC7567387; doi:10.1371/journal.pone.0240238)
Supplement: S1 Table — (DOCX) [file pone.0240238.s002.docx]

**S1 Table.** The basic information of the field-investigated.

| Transect | Site | Longitude/°E | Latitude/°N | Altitude/m | MAT/℃ | MAP/mm | Aridity | Vegetation Type |
| --- | --- | --- | --- | --- | --- | --- | --- | --- |
| Mongolian | 01 | 123.5059 | 44.59317 | 144 | 5.09626 | 448.4711 | 0.0319 | Meadow |
| Plteau | 02 | 121.0365 | 44.51578 | 269 | 5.79732 | 384.3864 | 0.0268 | Meadow |
|  | 03 | 120.3319 | 45.10762 | 660 | 3.7154 | 384.4745 | 0.0293 | Meadow |
|  | 04 | 118.357 | 44.77291 | 1019 | 0.557572 | 403.5822 | 0.0289 | Steppe |
|  | 05 | 116.5243 | 44.26304 | 1129 | 1.17077 | 315.4872 | 0.023 | Steppe |
|  | 06 | 116.6718 | 43.54849 | 1272 | 0.161427 | 385.8081 | 0.0269 | Steppe |
|  | 07 | 117.6763 | 44.50519 | 1024 | 1.96493 | 344.6781 | 0.0268 | Steppe |
|  | 08 | 114.895 | 44.00557 | 1101 | 0.102255 | 281.1772 | 0.0173 | Desert |
|  | 09 | 113.5001 | 43.84221 | 1022 | 2.47122 | 213.1613 | 0.0135 | Desert |
|  | 10 | 112.1488 | 43.63334 | 955 | 3.69424 | 168.2948 | 0.0118 | Desert |
| Loess | 01 | 113.357 | 36.29156 | 804 | 11.8458 | 563.8754 | 0.0427 | Meadow |
| Plateau | 02 | 112.2941 | 35.99317 | 894 | 9.96021 | 591.1833 | 0.0398 | Meadow |
|  | 03 | 111.635 | 35.98536 | 833 | 10.6581 | 566.1217 | 0.0364 | Meadow |
|  | 04 | 110.1814 | 36.07281 | 966 | 10.7233 | 533.3625 | 0.0367 | Steppe |
|  | 05 | 109.2421 | 36.7439 | 1268 | 9.49594 | 498.8911 | 0.0354 | Steppe |
|  | 06 | 107.9219 | 36.92894 | 1383 | 7.46464 | 438.0895 | 0.0323 | Steppe |
|  | 07 | 107.1862 | 37.57512 | 1535 | 5.22609 | 395.1434 | 0.0243 | Steppe |
|  | 08 | 105.7775 | 37.42154 | 1293 | 5.86873 | 320.3482 | 0.0149 | Desert |
|  | 09 | 104.9245 | 37.44234 | 1378 | 7.55535 | 233.8788 | 0.013 | Desert |
|  | 10 | 104.4444 | 37.46445 | 1714 | 7.70569 | 215.5 | 0.0128 | Desert |
| Tibetan | 01 | 95.45476 | 31.46381 | 4104 | 0.409005 | 619.8649 | 0.0607 | Meadow |
| Plateau | 02 | 93.53015 | 31.85132 | 4509 | -1.50039 | 536.7682 | 0.0639 | Meadow |
|  | 03 | 92.01396 | 31.64385 | 4587 | -4.3713 | 501.3833 | 0.0386 | Meadow |
|  | 04 | 90.74223 | 31.38466 | 4617 | -6.75953 | 526.8914 | 0.0348 | Steppe |
|  | 05 | 89.72158 | 31.54224 | 4588 | -3.05951 | 443.7926 | 0.0333 | Steppe |
|  | 06 | 87.82383 | 31.87083 | 4570 | -2.5676 | 389.0946 | 0.0208 | Steppe |
|  | 07 | 85.83923 | 31.92051 | 4938 | -3.76746 | 376.1719 | 0.0101 | Steppe |
|  | 08 | 83.34302 | 32.4138 | 4578 | -3.9025 | 317.1327 | 0.0056 | Desert |
|  | 09 | 81.23439 | 32.29886 | 4558 | -3.49254 | 291.4214 | 0.0079 | Desert |
|  | 10 | 80.14526 | 32.48434 | 4328 | -1.27199 | 191.7089 | 0.0066 | Desert |
